# Tcf3 Represses Wnt–β-Catenin Signaling and Maintains Neural Stem Cell Population during Neocortical Development

**DOI:** 10.1371/journal.pone.0094408

**Published:** 2014-05-15

**Authors:** Atsushi Kuwahara, Hiroshi Sakai, Yuanjiang Xu, Yasuhiro Itoh, Yusuke Hirabayashi, Yukiko Gotoh

**Affiliations:** Laboratory of Cell Signaling, Institute of Molecular and Cellular Biosciences, The University of Tokyo, Tokyo, Japan; National Cancer Institute, United States of America

## Abstract

During mouse neocortical development, the Wnt–β-catenin signaling pathway plays essential roles in various phenomena including neuronal differentiation and proliferation of neural precursor cells (NPCs). Production of the appropriate number of neurons without depletion of the NPC population requires precise regulation of the balance between differentiation and maintenance of NPCs. However, the mechanism that suppresses Wnt signaling to prevent premature neuronal differentiation of NPCs is poorly understood. We now show that the HMG box transcription factor Tcf3 (also known as Tcf7l1) contributes to this mechanism. Tcf3 is highly expressed in undifferentiated NPCs in the mouse neocortex, and its expression is reduced in intermediate neuronal progenitors (INPs) committed to the neuronal fate. We found Tcf3 to be a repressor of Wnt signaling in neocortical NPCs in a reporter gene assay. Tcf3 bound to the promoter of the proneural bHLH gene *Neurogenin1 (Neurog1)* and repressed its expression. Consistent with this, Tcf3 repressed neuronal differentiation and increased the self-renewal activity of NPCs. We also found that Wnt signal stimulation reduces the level of Tcf3, and increases those of Tcf1 (also known as Tcf7) and Lef1, positive mediators of Wnt signaling, in NPCs. Together, these results suggest that Tcf3 antagonizes Wnt signaling in NPCs, thereby maintaining their undifferentiated state in the neocortex and that Wnt signaling promotes the transition from Tcf3-mediated repression to Tcf1/Lef1-mediated enhancement of Wnt signaling, constituting a positive feedback loop that facilitates neuronal differentiation.

## Introduction

The canonical Wnt–β-catenin signaling pathway has a variety of roles in stem cell regulation during development and throughout adult life, ranging from maintenance of multipotency to induction of fate commitment [1], [2]. Neural precursor cells (NPCs) in the mammalian central nervous system are multipotent tissue stem cells that sequentially generate neurons and glial cells during development [3]–[5]. The Wnt–β-catenin pathway is active in the neocortical ventricular zone (VZ), where NPCs reside and start to differentiate, and plays critical roles in regulating proliferation of neocortical NPCs [6]–[10]. During the neurogenic phase of neocortical development, the Wnt–β-catenin pathway also induces neuronal differentiation of NPCs and intermediate neuronal progenitors (INPs) in part through expression and activation of Neurogenin1 (Neurog1), Neurogenin2 (Neurog2) and N-myc genes [7], [11]–[15]. Since the balance between maintenance and differentiation of NPCs is essential for generating an appropriate number of neurons and for establishing the fine brain architecture, the activity of the Wnt–β-catenin pathway should be precisely regulated. In particular, precocious (or excess) activation of this pathway should be suppressed to avoid, for example, premature neurogenesis. Although many extracellular and intracellular molecules have been identified to regulate the Wnt–β-catenin pathway, how the activity of this pathway is controlled in NPCs is still largely unknown.

Activation of the Wnt–β-catenin pathway results in the stabilization of β-catenin, which in turn associates with members of the Tcf/Lef family of DNA binding proteins and induces transcription of their target genes [1]. The Tcf/Lef family proteins contain the high-mobility group (HMG) DNA-binding domain and the β-catenin binding domain. In the absence of β-catenin binding, they function as transcriptional repressors, and β-catenin binding converts them into transcriptional activators [16], [17]. In mammals, the Tcf/Lef family comprises four members Tcf1, Lef1, Tcf3 and Tcf4 (also known as Tcf7l2) with various isoforms, which appear to be functionally specialized [18]–[20]. Whereas Lef1 and Tcf1 are required for transcriptional activation of the Wnt target genes, Tcf3 functions predominantly as a transcriptional repressor that acts independently of β-catenin binding [21]–[24] (with some exceptions: [25]). Intriguingly, Tcf3 was found to be expressed in different types of stem cells including embryonic and hair follicle stem cells. Whereas Tcf3 promotes differentiation of ES cells, in part through counteracting Wnt-mediated maintenance signals, it promotes the maintenance of hair follicle stem cells, in part through counteracting Wnt-mediated epidermal differentiation [26], [27]. A recent study reported that Tcf3 is also expressed in the neocortical VZ and that Tcf3 overexpression suppresses and knockdown promotes neuronal differentiation of neocortical NPCs [28]. Although it was proposed in this study (Ohtsuka et al.) that Tcf3 positively mediates an anti-neurogenic function of Wnt signaling, it remains unclear whether Tcf3 suppresses a neurogenic function of Wnt signaling or promotes an anti-neurogenic function of Wnt signaling, and which cell types in the VZ express Tcf3. Furthermore, it has remained elusive whether (and how) Tcf3 is regulated in the neocortical VZ. In this study, we found that Tcf3 is specifically expressed in an undifferentiated population of NPCs in the VZ. We also found that Tcf3 suppresses Wnt signaling and counteracts Wnt-mediated neuronal differentiation of NPCs. Our results thus indicate that Tcf3 serves as a transcriptional repressor that maintains the undifferentiated NPC population in the developing neocortex. Our results also suggest that the activation of Wnt signaling reduces the level of Tcf3 and increases those of full-length Lef1 and Tcf1 isoforms in neocortical NPCs, which may enhance the responsiveness to Wnt signaling and thus accelerate neuronal differentiation of NPCs.

## Materials and Methods

### Ethics Statement and Animals

All animal experiments were performed in accordance with the protocol approved by the Animal Care and Use Committee of the University of Tokyo. Pregnant ICR mice were obtained from CLEA Japan (Tokyo, Japan) and Oriental Yeast Co. (Tokyo, Japan). Nestin-d4-Venus transgenic mouse line was kindly provided by Drs. Sunabori T. and Okano H. [29]. The day of the vaginal plug detection was designated as embryonic day 0.5 (E0.5).

### Vectors and Transfection

The plasmids pMX-EGFP (pMX-GFP) and pMXs-IRES-EGFP (pMXs-IG) were kindly provided by Dr. T. Kitamura. The plasmids Super8×TOP-FLASH (8×TOP-FLASH) and Super8×FOP-FLASH (8×FOP-FLASH) were kindly provided by Dr. Randall T Moon [30]. Tcf3 (NM_009332), Tcf1 (ENSMUST00000072425) and Lef1 (NM_010703.3) were cloned from mouse developing neocortex cDNA library and subcloned into pMXs-IG and pCAG-IG. ΔN-Tcf3, an N-terminal truncated form of mouse Tcf3, and Tcf3-ΔHMG, a HMG box truncated form of mouse Tcf3, were amplified by PCR as described previously and subcloned into pMXs-IG and pCAG-IG [26]. The plasmids pSIREN-con (control shRNA), pSIREN-Luc (control shRNA) and pSIREN-Tcf3 #1,2 (Tcf3 shRNA #1,2) were generated in accordance with the manufacturers’ instructions (BD Biosciences and Clontech). Recombinant retroviruses were produced using the pMX vectors and pSIREN vectors as described previously [31]. Transfection of primary NPCs with pMXs, pCAG and pSIREN vectors were carried out by using Lipofectamine 2000 (Invitrogen).

### Antibodies

Antibodies used in this study were: goat antibodies to Tcf3 (#1, M-20, Santa Cruz) 1∶1000, Neurog1 (A-20, Santa Cruz) 1∶200, Neurog2 (Santa Cruz) 1∶500, mouse antibodies to βIII-tubulin (TuJ1, Covance) 1∶1000, Tcf3/4 (#2, Upstate) 1∶1000, Sox2 (R&D) 1∶100, chick antibody to GFP (Abcam) 1∶2000, and rabbit antibodies to GFP (MBL) 1∶1000, Pax6 (Chemicon) 1∶1000, Tbr2 (Chemicon) 1∶1000. Alexa-labeled secondary antibodies, TO-PRO-3 and Hoechst 33342 (for nuclear staining) were from Molecular Probes.

### Immunohistochemistry, RNA Probes and In situ Hybridization

Mouse embryos were fixed at 4°C in 4% paraformaldehyde in PBS for 0.5–12 h for immunohistochemistry (IHC) or overnight for in situ hybridization (ISH). Samples were cryoprotected overnight in 30% sucrose in PBS, embedded in OCT (Tissue-Tek), and frozen on dry ice. Frozen embryos were sectioned on a cryostat at 10–14 µm. Sections were processed for IHC or ISH as described [31], [32]. For ISH, PCR fragments with T3 or T7 promoter were amplified with specific primers for mouse Tcf3. Primers are listed in Supporting information. Digoxigenin (DIG)-labeled cRNA probes were transcribed from PCR fragments using the DIG RNA labeling mix (Roche) and T3 or T7 RNA polymerase (Promega or Takara, respectively) according to the manufacturers’ instructions. Frozen sections were rinsed in PBS containing 0.1% Tween-20 (PBS-T), digested with proteinase K at 37°C for 5 min, rinsed in PBS-T, post-fixed in 4% paraformaldehyde at room temperature for 20 min, and rinsed. Sections were hybridized at 65°C for about 16 h in a hybridization buffer containing 50% deionized formamide, 5×standard saline citrate (SSC, 150 mM sodium chloride and 15 mM sodium citrate for 1×SSC; pH 4.5), 1% SDS, 50 µg/ml yeast tRNA, 50 µg/ml heparin, and DIG-labeled cRNA probe. Sections were washed twice in 50% formamide, 5×SSC, 1% SDS at 65°C for 30 min, and subsequently twice with 50% formamide, 2×SSC at 65°C for 45 min. After washing with TBS containing 0.1% Tween-20 (TBS-T), sections were incubated for 1 h in a blocking solution (0.5% blocking reagent (Roche) in TBS-T), and then incubated with an alkaline phosphatase (AP)-coupled antibody (Roche) at 4°C overnight, rinsed, and processed for AP activity with NBT/BCIP (Wako).

### Primary NPC Culture, Neurosphere Assay, FACS, Immunocytochemistry, Western Blotting, Reporter Assay and ChIP Assay

Neocortical NPCs were isolated from the dosal cerebral cortex of mouse embryos at E11.5 and cultured in DMEM-F12 (Gibco) supplemented with 2% B27 (Gibco) and with or without FGF2 (20 ng/ml, Invitrogen) and EGF (20 ng/ml, Upstate). Mouse Wnt3a protein, purified as described previously [7], [33], was used for experiments at a final concentration of 0.50 µg/ml. The vehicle (2.5 mM NaCl, 0.005% CHAPS) was used as a control of Wnt treatment. CHIR99021 was purchased from Wako Pure Chemical Industries and used for experiments at a final concentration of 10 µM. Neurosphere assay, immunocytochemistry, Western blotting, reporter assay and ChIP assay were performed as described previously [7], [11], [31]. FACS was performed as described previously [29]. In brief, dissociated neocortical cells from E14.5 transgenic or wild-type mouse embryos were analyzed and sorted by using AriaIII (BD) with DIVA software.

### RNA Extraction, Northern Blotting and quantitative PCR (qPCR) Analysis

RNA extraction was carried out using RNAiso (Takara) following the instructions of the manufacturer. Extracted RNAs were treated with DNaseI (Takara) to remove contaminating genomic DNA, and further purified using RNAiso. Total RNA was further purified by poly-A selection in some experiments. Total RNA and poly-A selected RNA were analyzed by Northern blotting using DIG-labeled cRNA probe according to the manufacturers’ instructions (Roche). Reverse transcription (RT) was performed with 2 µg of total RNA, oligo dT (20 mer) and ReverTra Ace (TOYOBO). The resulting cDNA was subjected to qPCR in a Roche LightCycler with SYBR *Premix Ex Taq* (Takara). Primers are listed in Text S1.

### Statistical Analysis

A statistical analysis was performed using the unpaired two-tailed Student’s *t*-test between control and experimental conditions. A *P* value of <0.05 was considered statistically significant. *P<0.05, **P<0.01, ***P<0.001 for all figures.

## Results

### Tcf3 is Expressed in Pax6^+^ NPCs in the Developing Neocortex

**Figure 1:**
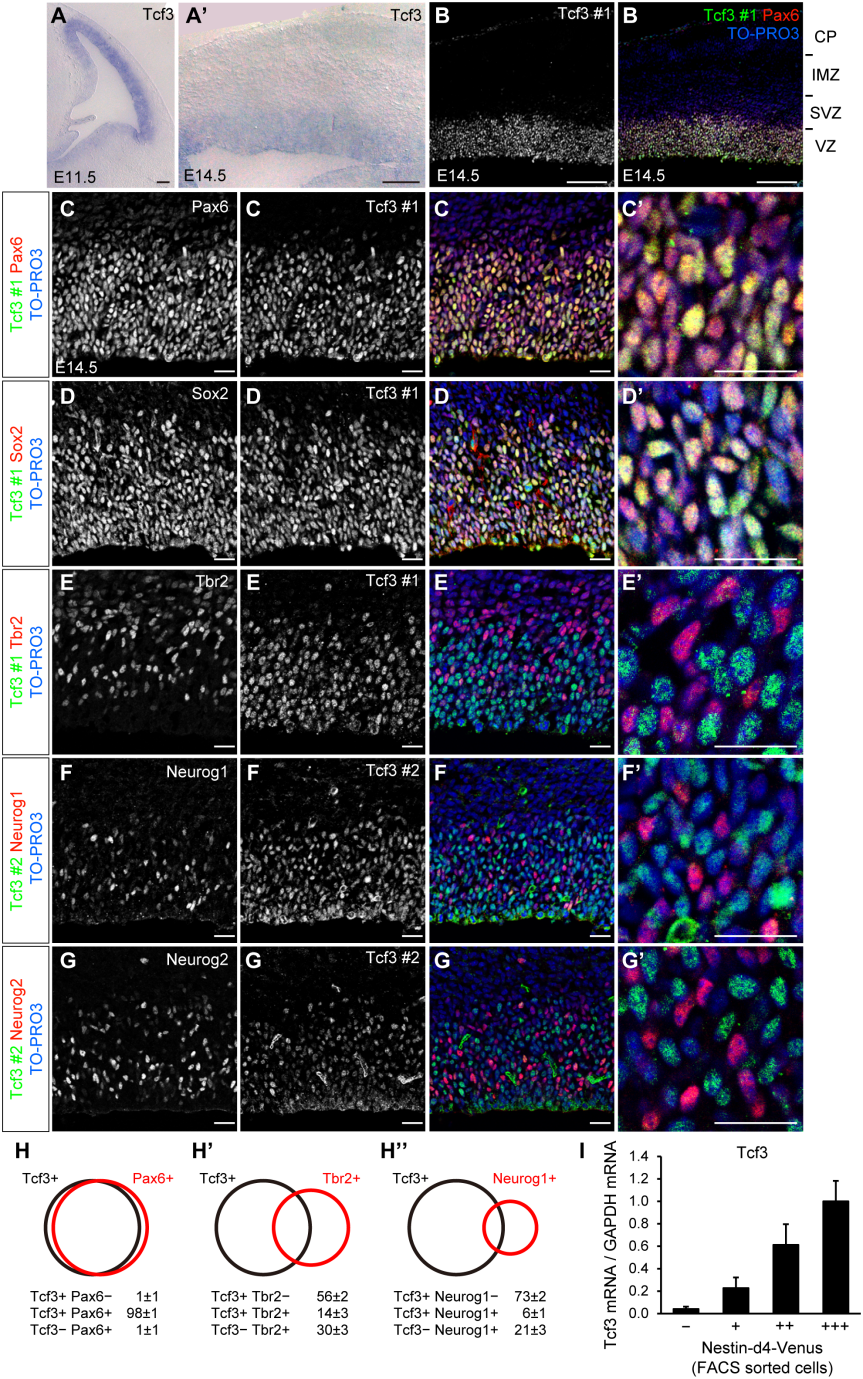
Tcf3 is expressed in Pax6^+^ NPCs in the developing neocortex. ***A,A’*** *,* In situ hybridization of coronal sections of the E11.5 (***A***) and E14.5 (***A’***) mouse cortex for the Tcf3 mRNA. ***B–H”***, Coronal sections of E14.5 neocortex immunostained as indicated. VZ, ventricular zone; SVZ, subventricular zone; IMZ, intermediate zone; CP, cortical plate (***B***). Higher magnification of VZ in ***C–G*** are shown in ***C’–G’***. Scale bars, 100 µm (***A,A’,B***) and 20 µm (***C–G’***). The percentages of Tcf3^+^Pax6^−^ cells, Tcf3^+^Pax6^+^ cells and Tcf3^−^Pax6^+^ cells among either Tcf3^+^ or Pax6^+^ cells in the VZ were determined by immunostaining (***H*** *,* below). The percentages of Tcf3^+^Tbr2^−^ cells, Tcf3^+^Tbr2^+^ cells and Tcf3^−^Tbr2^+^ cells and those of Tcf3^+^Neurog1^−^ cells, Tcf3^+^Neurog1^+^ cells and Tcf3^−^Neurog1^+^ cells are shown in ***H’,H”***. Venn diagram of these percentages (***H-H”***, above). ***I***, dissociated cells from E14.5 neocortices of Nestin-d4-Venus transgenic mouse were sorted into Nestin-d4-Venus -, Nestin-d4-Venus +, Nestin-d4-Venus ++, and Nestin-d4-Venus +++ fractions by using FACS (see also Fig. S1). The mRNA level of Tcf3 in each fractions was determined by qPCR analysis. Data represents mean ± SEM (***H-H”, I***).

We investigated the expression patterns of Tcf3 in the developing neocortex as a candidate regulator of Wnt signaling. First we performed in situ hybridization analysis. We found Tcf3 mRNA was specifically expressed in the VZ but not in the intermediate zone (IMZ) or the cortical plate (CP) of the mouse neocortex at E11.5 and E14.5 (Fig. 1A,A’), consistent with previous reports [28], [34], [35]. Since the neocortical VZ contains undifferentiated NPCs (also called radial glia) and differentiating neurons (that include intermediate neuronal progenitors (INPs) and postmitotic neurons), we examined which of these cell populations expresses Tcf3. In the neocortex at E14.5, most Tcf3 positive cells also expressed Pax6 and Sox2, markers for undifferentiated NPCs, and most Pax6 and Sox2 positive cells expressed Tcf3 as revealed by an immunohistochemical analysis (Fig. 1B–D’,H). In contrast, Tcf3 was not expressed in cells expressing Neurog1, Neurog2 (markers for cells biased toward neuronal fate commitment) or Tbr2 (a marker for INPs and newborn neurons) in the neocortical VZ (Fig. 1E–G’,H’,H”). Further, we isolated undifferentiated and differentiated cells from Nestin-d4-Venus transgenic mouse at E14.5 by FACS. We found that the expression of Tcf3 mRNA was high in Nestin-d4-Venus strong positive undifferentiated NPC fraction, but reduced in more differentiated cell fractions with lower Nestin-d4-Venus intensities (Fig. 1I, Fig. S1) [29]. These results suggest that Tcf3 is expressed in undifferentiated NPCs but disappears in cells committed to the neuronal fate in the developing neocortical VZ.

### Tcf3 Represses Wnt Signaling in Neocortical NPC Cultures

**Figure 2:**
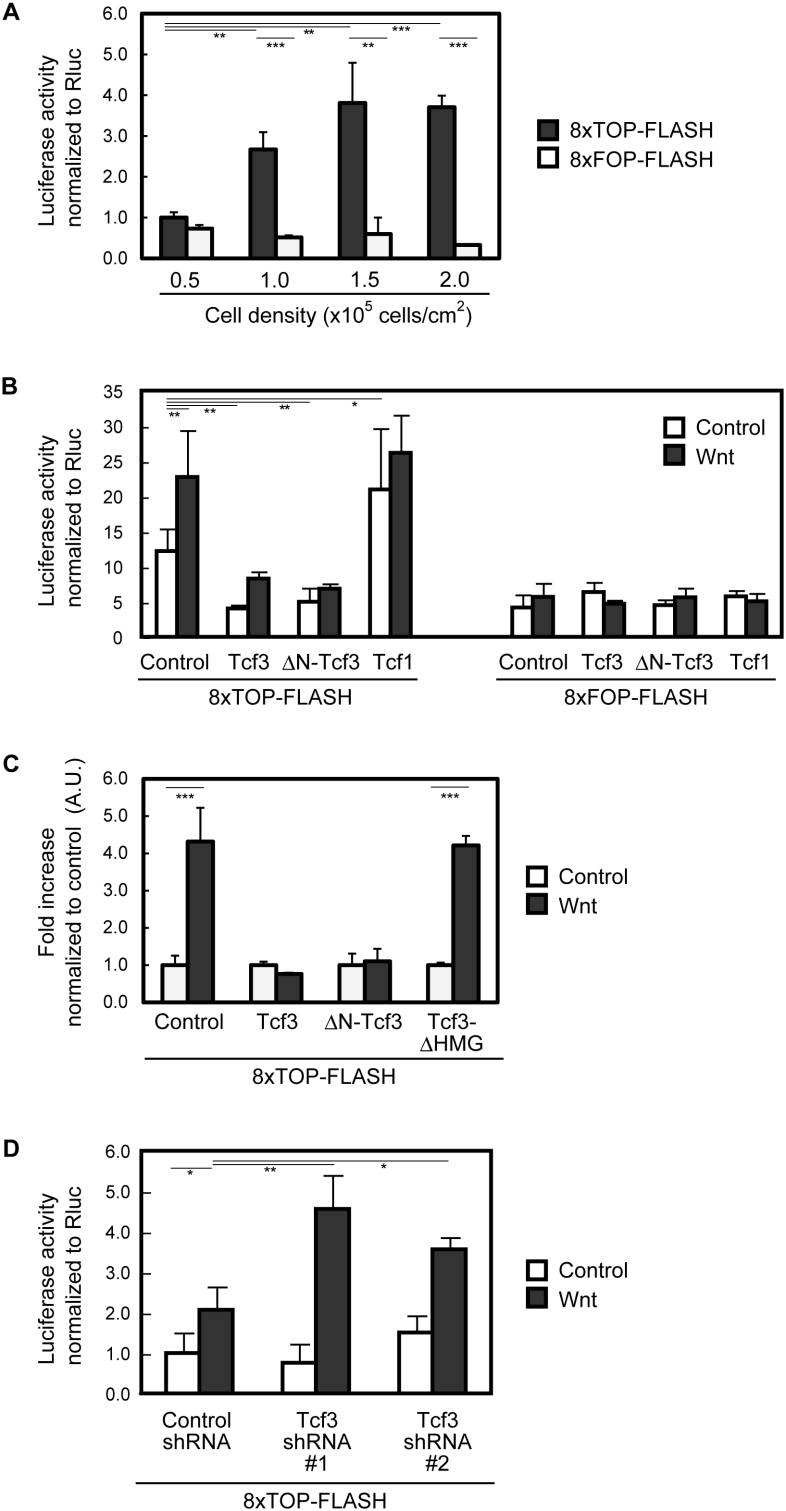
Tcf3 represses β-catenin reporter activity in NPCs. ***A***, NPCs isolated from E11.5 neocortex were plated at 0.5–2.0×10^5^ cells/cm^2^ on PDL-coated dishes and incubated with FGF2. The cells were transfected with β-catenin reporter (8×TOP-FLASH) or their mutant reporter (8×FOP-FLASH, see text) and cultured in the presence of FGF2 for 2d. Cells were subjected to the luciferase assay normalized with internal control (pGL3-TK-Rluc). Data represents the mean ± SD of three independent samples. ***B***, NPCs isolated from E11.5 neocortex were cultured in suspension for 3 d with FGF2 and EGF. The cells were dissociated and plated on PDL-coated dishes, and incubated with FGF2. The cells were transfected with β-catenin reporter (8×TOP-FLASH) or their mutant reporter (8×FOP-FLASH) together with control, Tcf3, ΔN-Tcf3 (an N-terminal truncated form) or Tcf1, and then treated with recombinant Wnt3a or vehicle control for 4 h in the presence of FGF2. Cells were subjected to the luciferase assay as ***A***. ***C***, E11.5 NPCs were acutely plated on PDL-coated dishes in the presence of FGF2, and transfected with β-catenin reporter (8×TOP-FLASH) together with control, Tcf3, ΔN-Tcf3 or Tcf3-ΔHMG (a DNA binding domain truncated form, see text), and then treated with recombinant Wnt3a or vehicle control for 4 h in the presence of FGF2. Cells were subjected to the luciferase assay normalized with internal control (pGL3-TK-Rluc). Data represents fold increase normalized to vehicle control and the mean ± SD of three independent samples. ***D***, E11.5 NPCs were acutely plated on PDL-coated dishes in the presence of FGF2, and transfected with β-catenin reporter (8×TOP-FLASH) together with control, Tcf3 shRNA#1, or Tcf3 shRNA#2. After 2 d, the cells were treated with Wnt3a or control for 6 h in the presence of FGF2 and assayed for luciferase activity as ***A***.

We then asked whether Wnt signaling is active in neocortical NPCs. NPCs, acutely prepared from E11.5 mouse neocortices, were plated on poly-D-lysine (PDL) coated dishes and transfected with the reporter plasmids, and then cultured in the presence of FGF2 for 2 d. We found that the 8×TOP-FLASH reporter (TOP-FLASH), which harbors 8 repeats of the consensus binding sequence for Tcf/Lef family proteins (ATCAAAGC) upstream of the basal promoter, exhibited significantly higher activity than the 8×FOP-FLASH reporter (FOP-FLASH), in which each Tcf binding sequence is replaced by a mutant sequence (gcCAAAGC) (Fig. 2A). Importantly, the activity of TOP-FLASH was increased in a cell density dependent manner. These data suggest that endogenous Wnt signaling is active in these NPCs, which is consistent with previous reports using reporter mice that suggest that Wnt signaling is activated in the neocortical NPCs in vivo [36], [37].

Wnt signaling acts in the developing neocortex in a stage, cell type and context-dependent manner. Whereas it has been established that Wnt signaling promotes proliferation of NPCs, the role of Wnt signaling in promoting or suppressing neuronal differentiation of neocortical NPCs has been controversial. A recent report showed that overexpression of Tcf3 by in utero electroporation suppressed neuronal differentiation of neocortical NPCs, and knockdown of Tcf3 had an opposite effect [28]. Despite Tcf3 might have a potential to promote gene transcription induced by Wnt stimulation in some contexts as Ohtsuka et al discussed, Tcf3 functions as a transcriptional repressor in most in vivo contexts [19], [21]–[24]. We thus wanted to know whether this result reflects a role of Tcf3 in enhancing the anti-neurogenic function of Wnt signaling or inhibiting the pro-neurogenic function of Wnt signaling. Therefore, we used a reporter gene assay to examine whether Tcf3 enhances or suppresses Wnt signaling in the neocortical NPC culture. Undifferentiated NPCs were enriched among cells collected from E11.5 neocortices by culturing in suspension in the presence of FGF2 and EGF for 3 days (E11.5+3 days in vitro (div) culture) and then transfected with expression plasmids and the reporter plasmids. Treatment of the E11.5+3 div culture with recombinant Wnt3a increased the activity of TOP-FLASH, but not that of FOP-FLASH (Fig. 2B). We found that overexpression of Tcf3 repressed the activity of TOP-FLASH in the presence or absence of Wnt3a stimulation. In contrast, expression of the full-length Tcf1, a positive regulator of Wnt signaling [18], [20], [24], [38], did not repress, and rather enhanced, the activity of TOP-FLASH in these cells. Expression of an N-terminal truncated form of Tcf3 (ΔN-Tcf3), a constitutive repressor form of Tcf3, also repressed the activity of TOP-FLASH in both control and Wnt-treated NPCs to a similar extent with the full-length Tcf3 (Fig. 2B). Importantly, expression of an HMG domain truncated form of Tcf3 (Tcf3-ΔHMG), a DNA-binding domain mutant of Tcf3, did not repress the Wnt induced increase of TOP-FLASH activity (Fig. 2C). These results indicate that Tcf3 can function as a repressor for Wnt signaling when overexpressed in undifferentiated neocortical NPCs. A similar observation was reported in a recent study [34].

We then examined whether endogenous Tcf3 also functions as a repressor of Wnt signaling in NPCs. Expression of two different short hairpin RNA (shRNA) constructs targeting Tcf3 (shTcf3#1 and #2) reduced the level of Tcf3 mRNA in NPCs (data not shown). This knockdown of Tcf3 significantly enhanced the activation of TOP-FLASH by Wnt treatment (Fig. 2D). This suggests that endogenous Tcf3 indeed suppresses the target activation by Wnt signaling.

### Tcf3 Represses Wnt-stimulated Neuronal Differentiation

**Figure 3:**
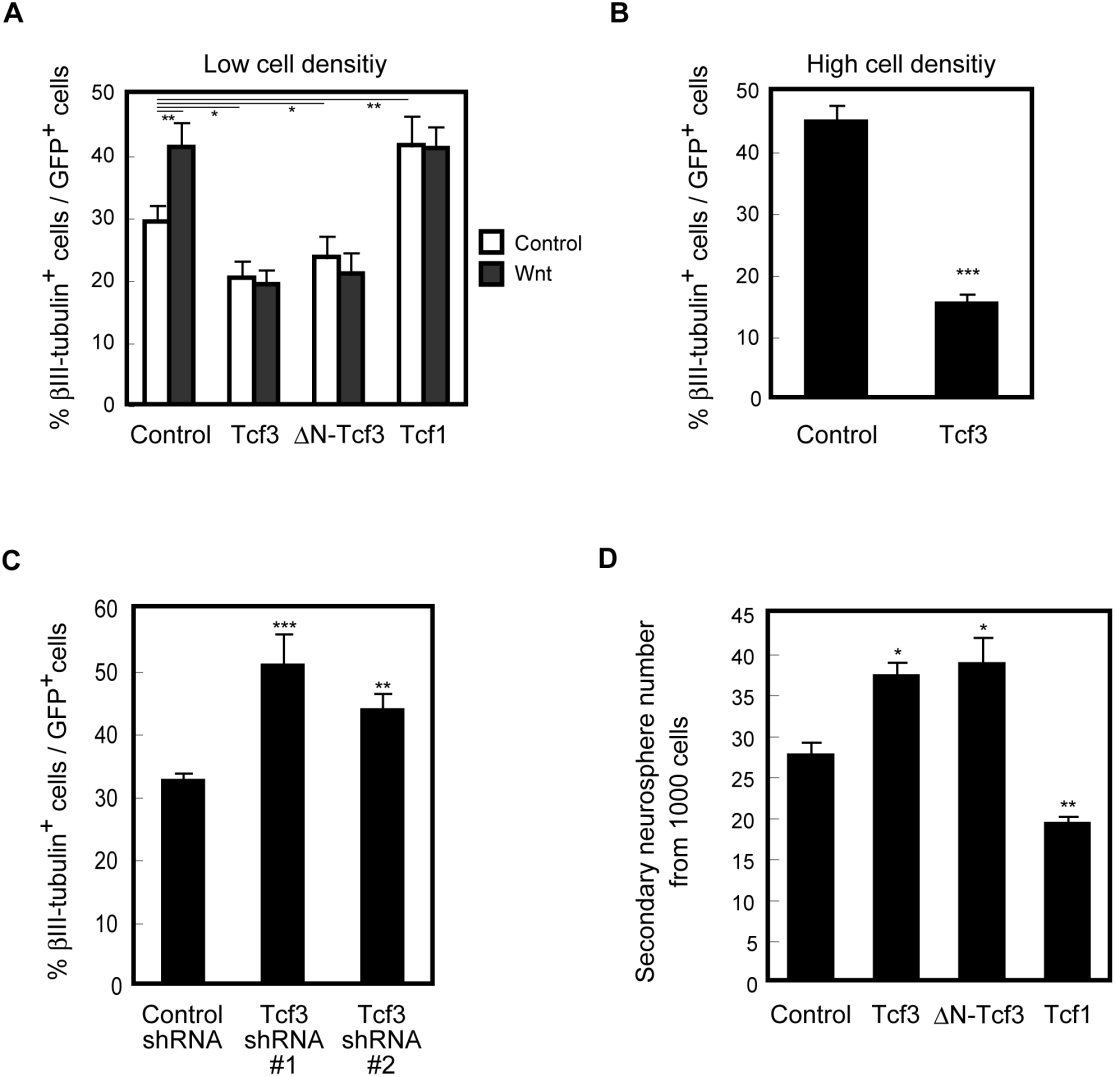
Tcf3 increases neurosphere-forming activity and represses neuronal differentiation of NPCs. ***A***, E11.5 NPCs were plated at 1.0×10^5^ cells/cm^2^ with FGF2 and infected with a retrovirus encoding GFP alone (control), both GFP and Tcf3 (Tcf3), both GFP and ΔN-Tcf3 (ΔN-Tcf3) or both GFP and Tcf1 (Tcf1) and incubated for 1 d with FGF2. The cells were trypsinized and replated at 0.66×10^5^ cells/cm^2^ cells and incubated for 2 d with FGF2 (undifferentiated condition), and then treated with Wnt3a or control for 24 h in the presence of FGF2 and cultured for another 2 d without FGF2 to induce differentiation. The percentage of βIII-tubulin^+^ cells among GFP^+^ cells was determined by immunostaining. ***B***, E11.5 NPCs were plated at 1.0×10^5^ cells/cm^2^ with FGF2 and infected with control or Tcf3. The cells were incubated for 3 d with FGF2 (undifferentiated condition) and for another 2 d without FGF2 to induce differentiation. The percentage of βIII-tubulin^+^ cells among GFP^+^ cells was determined by immunostaining. ***C***, E11.5 NPCs were cultured for 1 d with FGF2 and infected with a retrovirus encoding GFP and Luc-shRNA (control), both GFP and Tcf3-shRNA #1 (Tcf3 shRNA #1), or GFP and Tcf3-shRNA #2 (Tcf3 shRNA #2). Then the cells were incubated for 3 d with FGF2 and for another 2 d without FGF2 to induce differentiation. The percentage of βIII-tubulin^+^ cells among GFP^+^ cells was determined by immunostaining. ***D***, E11.5 NPCs were infected with a retrovirus encoding control, Tcf3, ΔN-Tcf3 or Tcf1 and incubated in suspension culture for 4 d with FGF2 (primary sphere). Cells were plated in suspension at a low density and incubated for 7 d in the presence of FGF2 (secondary sphere). Data represent the number of formed cell aggregates (neurospheres). ***A–D***, Data represents mean ± SEM (***A–C***) and mean ± SD (***D***).

Given that Tcf3 suppresses neuronal differentiation in the developing neocortex [28] and that Tcf3 functions as a transcriptional repressor of Wnt signaling in the neocortical NPC culture (Fig. 2) [34], we hypothesized that Tcf3 antagonizes the neurogenic function of Wnt signaling in these neocortical NPCs. We then investigated whether Tcf3 also represses neuronal differentiation in the neocortical NPC cultures in the presence or absence of Wnt3a treatment. We infected E11.5 neocortical cultures with retroviruses harboring Tcf3, ΔN-Tcf3 or Tcf1 and cultured them for 4 days before treating them with recombinant Wnt3a. We found that overexpression of Tcf3 indeed reduced the percentage of infected (GFP^+^) cells that were positive for the neuronal marker βIII-tubulin, under a differentiation-inducing condition (culturing in the absence of growth factors) (Fig. 3A). Interestingly, this Tcf3-mediated suppression of neuronal differentiation was more prominent in high density cultures than in low density cultures (Fig. 3A and B), possibly reflecting a greater activation of endogenous Wnt signaling in high density cultures (Fig. 2A) [39]. Importantly, the treatment of low density NPC cultures with recombinant Wnt3a increased the percentage of βIII-tubulin-positive cells among GFP-positive cells and this increase was canceled by Tcf3 overexpression. This effect of Tcf3 appears to be due to its function as a transcriptional repressor and independent of its β-catenin binding, since overexpression of ΔN-Tcf3, which lacks the β-catenin binding domain, had similar effects on Wnt-enhanced neuronal differentiation. Conversely, knockdown of endogenous Tcf3 by infecting retroviruses encoding shRNA targeting Tcf3 significantly enhanced the percentage of βIII-tubulin-positive cells among infected (GFP^+^) cells (Fig. 3C). These results suggest that Wnt signaling enhances neuronal differentiation in this NPC culture and that Tcf3 suppresses this function of Wnt signaling. We then examined the effect of the full-length Tcf1, which enhanced the endogenous activity of Wnt signaling. We found that overexpression of Tcf1 increased the percentage of βIII-tubulin-positive cells among GFP-positive cells in the absence of Wnt3a treatment (Fig. 3A). These support the notion that Wnt signaling promotes neuronal differentiation and expression of Tcf family proteins regulates this effect.

To investigate whether Tcf3 maintains the undifferentiated state of NPCs by suppressing neuronal differentiation, we carried out a neurosphere assay, which is commonly used for monitoring the amount of undifferentiated NPCs. We infected E11.5 neocortical cultures with retroviruses harboring Tcf3, ΔN-Tcf3 or Tcf1 and cultured them for 4 days in suspension (primary neurospheres). These cells were then dissociated completely and cultured in suspension at a very low (clonal) density (1000 cells/well in a 96 well dish) (secondary neurospheres). We found that the number of secondary neurospheres formed from 1000 cells obtained from primary neurospheres was significantly higher in Tcf3 or ΔN-Tcf3-expressing cultures compared with control cultures (Fig. 3D). These results suggest that expression of Tcf3 promotes the maintenance of undifferentiated neocortical NPCs. On the other hand, the expression of Tcf1 reduced the number of secondary neurospheres, consistent with the idea that Tcf1 enhances neuronal differentiation.

### Tcf3 Directly Suppresses Neurog1 and N-myc Expression

**Figure 4:**
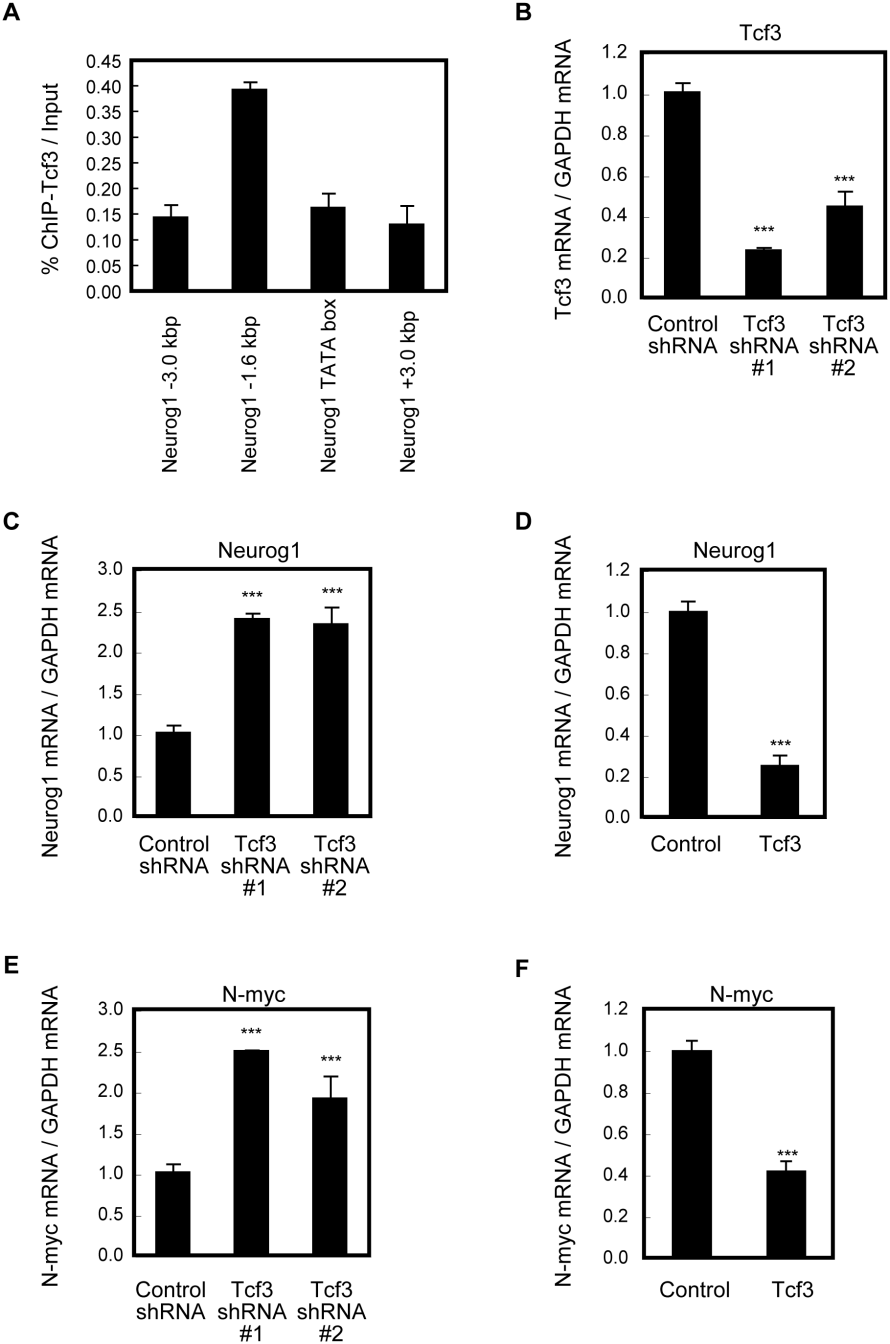
Tcf3 directly represses transcription of Neurog1 and N-myc. ***A***, Chromatin complex was immunoprecipitated from E11.5 neocortical lysates with anti-Tcf3. The immunoprecipitates were subjected to qPCR analysis. ***B,C***, NPCs were infected with a retrovirus encoding control, Tcf3 shRNA #1 or Tcf3 shRNA #2 and incubated with FGF2 for 3 d. Cells were cultured for another 6 h in the presence (undifferentiated condition) or absence (differentiated condition) of FGF2. The mRNA levels of Tcf3 (***B***) and Neurog1 (***C***) were determined by qPCR analysis. Data obtained in differentiated condition are shown in ***B,C***. Similar results were obtained in undifferentiated condition (not shown). ***D***, E11.5 NPCs were infected with a retrovirus encoding control or Tcf3 and incubated with FGF2 for 3 d. The level of Neurog1 mRNA was determined by qPCR analysis. ***E,F***, NPCs were infected with a retrovirus encoding control, Tcf3 shRNA #1 (***E***), Tcf3 shRNA #2 (***E***) or Tcf3 (***F***) as ***C,D***. Then the cells were incubated in the presence of FGF2 for 3 d. The level of N-myc mRNA was determined by qPCR analysis. ***B–F***, Data are normalized with GAPDH mRNA (arbitrary unit). ***A–F***, Data represents mean ± SEM.

Our observations that most cells expressing Neurog1 in the neocortical VZ were devoid of Tcf3 (Fig. 1F,F’,H”) and that Wnt signaling directly regulates the promoter of the proneural gene Neurog1 through a functional Tcf/Lef binding sequence [11] prompted us to investigate whether Neurog1 might be a direct target for Tcf3 in suppressing the neuronal differentiation of neocortical NPCs. We carried out a chromatin immunoprecipitation (ChIP) assay with an antibody to Tcf3 in E11.5 neocortex and found that Tcf3 protein was indeed enriched around the Tcf/Lef binding site located at −1.6 kb of the transcription start site of the *Neurog1* gene (Fig. 4A). Furthermore, knockdown of endogenous Tcf3 by using shRNA against Tcf3 resulted in an increase in Neurog1 expression in E11.5 neocortical NPC cultures (Fig. 4B,C). Conversely, overexpression of Tcf3 reduced the expression of Neurog1 in these NPCs (Fig. 4D). Together, these results indicate that Tcf3 directly binds to the *Neurog1* locus and suppresses its expression.

We also examined the effect of Tcf3 on the expression of N-myc, since Tcf3 directly binds to the promoter of N-myc and the expression of ΔN-Tcf3 repressed the expression of N-myc [7], [40]. Strikingly, knockdown and overexpression of Tcf3 increased and decreased the expression of N-myc, respectively, but not that of Cyclin D1 (Fig. 4E,F and Fig. S2).

Since Neurog1 is a key determinant of the neuronal fate in the neocortex and N-myc is required for the proper production of INPs, suppression of Neurog1 and N-myc might underlie the anti-neurogenic role of Tcf3 at least in part.

### Wnt Signaling Reduces the Expression of Tcf3

**Figure 5:**
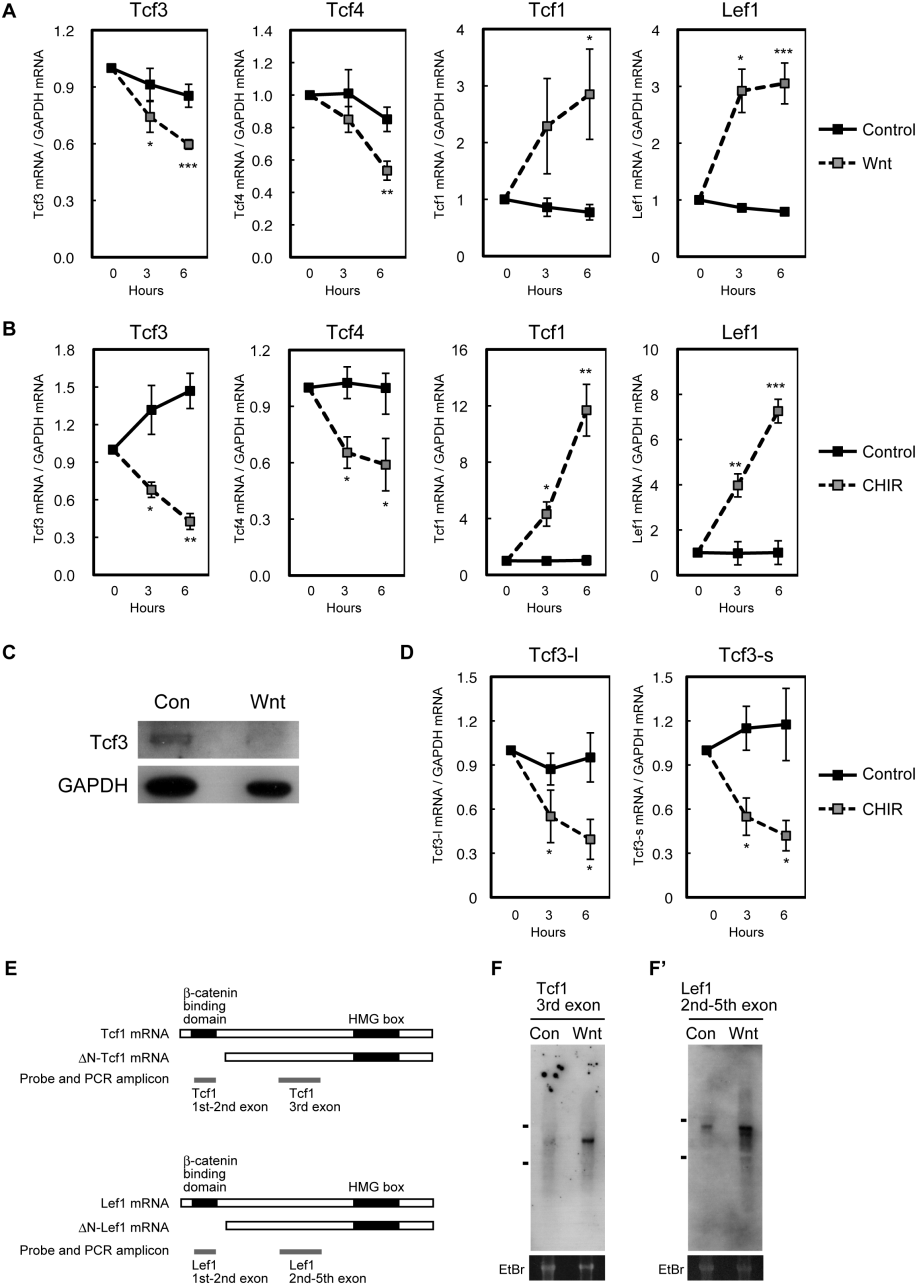
Wnt signaling decreases the expression of Tcf3 and increases that of Tcf1 and Lef1 containing β-catenin binding domain. ***A–B***, E11.5 NPCs were incubated in the presence of FGF2 with control, recombinant Wnt3a (***A***) or CHIR99021 (***B***) for 0, 3 or 6 h. The mRNA levels of Tcf3, Tcf4, Tcf1 (1st-2nd exon shown in ***E***) and Lef1 (1st-2nd exon shown in ***E***) were determined by qPCR analysis. Data are normalized with GAPDH mRNA (arbitrary unit) and represent the mean of three independent samples ± SD. ***C***, E11.5 NPCs were incubated in the presence of FGF2 with control or recombinant Wnt3a for 24 h. Cell lysates were subjected to Western blotting analysis with antibodies to Tcf3 and GAPDH. ***D***, E11.5 NPCs were incubated in the presence of FGF2 with control or CHIR99021 for 0, 3 or 6 h. The mRNA levels of Tcf3-l and Tcf3-s were determined by qPCR analysis as ***B***. ***E–F’***, E11.5 NPCs were incubated in the presence of FGF2 with control or recombinant Wnt3a for 4 h. Poly-A selected RNA was subjected to Northern blotting analysis with Tcf1 or Lef1 domain specific probes shown in ***E***. ***F,F’***, Markers represent 28S (upper) and 18S (lower) ribosomal RNA. Ethidium bromide (EtBr) staining is shown as loading control (lower panel).

Given that Tcf3 suppresses Wnt-mediated neuronal differentiation and promotes the maintenance of undifferentiated NPCs in the neocortex, mechanisms that control Tcf3 expression, if any, would affect the regulation of neuronal differentiation. Surprisingly, we found that Wnt signaling itself regulates the expression level of Tcf3 and Tcf4. Treatment of neocortical NPCs with recombinant Wnt3a or with the GSK3 inhibitor CHIR99021 [41], [42] for 3 or 6 h decreased the levels of Tcf3 mRNA and Tcf4 mRNA compared with that of control cells (Fig. 5A,B). We also analyzed the amount of Tcf3 protein by Western blotting and found that treatment with Wnt3a for 24 h decreased the amount of Tcf3 protein (Fig. 5C). A previous report showed that a short isoform (Tcf3-s) and a long isoform (Tcf3-l) of Tcf3 are expressed in ES cells. Since the expression of Tcf3-l is higher in ES cells than in embryoid bodies and the 14 amino acid sequence unique to Tcf3-l overlaps the Groucho binding domain, these two isoforms might have different roles and their expressions are differentially regulated [43]. Therefore, we examined the expression levels of these isoforms by using the primers which specifically detect Tcf3-l or Tcf3-s and found that both of these Tcf3 isoforms were expressed in neocortical NPCs and that activation of Wnt signaling by CHIR99021 treatment reduced the expression of both isoforms (Fig. 5D).

In contrast to the reduction of Tcf3, the levels of Tcf1 and Lef1 mRNA were markedly increased in response to Wnt3a treatment or treatment with the GSK3 inhibitor CHIR99021 for 3 or 6 h (Fig. 5A,B). Previous studies showed that Wnt signaling induces the expression of the repressor isoforms of Tcf1, which lack the β-catenin interaction domain, and that of both the repressor and activator isoforms of Lef1 in the other tissues [44]–[47]. Therefore, we performed Northern blotting to examine the isoforms of Tcf1 and Lef1 transcribed in response to Wnt signaling. We first hybridized polyA^+^ RNAs isolated from NPCs treated with or without Wnt3a, with probes from 3rd exon of Tcf1 and 2nd-5th exon of Lef1, which are contained in all the Tcf1 and Lef1 isoforms respectively. In the control cells, Tcf1 3rd exon probe detected a major transcript of 1600 bp, which corresponds to the length of the full-length Tcf1 (ENSMUST00000072425) (Fig. 5E,F). Lef1 2nd-5th exon probe also detected a major transcripts of 2500 bp, which corresponds to the length of the full-length form of Lef1 (NM_010703.3) (Fig. 5E,F’). We found that treatment with Wnt3a significantly increased the amount of these full length forms of Tcf1 and Lef1 (Fig. 5F,F’). Further, we carried out quantitative PCR with the primers designed for detecting sequences corresponding to β-catenin binding domain of Tcf1 (1st-2nd exon) or Lef1 (1st-2nd exon), and confirmed that Wnt3a treatment or the treatment with CHIR significantly increased the Tcf1 and Lef1 isoforms containing β-catenin binding domain (Fig. 5A,B). These results suggest that the major Tcf1 and Lef1 isoforms expressed in the neocortex are the full-length forms and their expression can be stimulated by Wnt signaling.

## Discussion

The roles of the canonical Wnt pathway in early patterning of the cortex [48] and in proliferation/self-renewal of NPCs/radial glia have been well-established [7]–[11], [14]. It remains, however, controversial whether this signaling pathway promotes or inhibits neuronal differentiation of NPCs. Previous studies have shown that expression of a Wnt ligand or active β-catenin promotes neuronal differentiation of NPCs and/or INPs in various regions at different stages [7], [11]–[13], [15], [49]–[54]. Expression of a VP16-fused (transcriptionally active) form of Lef1 or Tcf4 was also found to drive neuronal differentiation [15], [55]. Consistent with this neurogenic action of Wnt signaling, loss-of-function mutations of LRP6 or Ryk (a mediator of Wnt signaling) inhibit cortical neurogenesis, and the loss of APC (a negative regulator of Wnt signaling) in the neocortex increases neurons in the VZ [13], [14], [56]. On the other hand, forced activation of β-catenin in neocortical NPCs suppresses their neuronal differentiation and causes cortical malformation [8], and conversely, focal ablation of β-catenin or expression of β-catenin inhibitors promotes NPC cell cycle exit and neuronal differentiation [10], [57]. Although these results suggest that β-catenin suppresses neurogenesis, these effects of β-catenin might be partly due to its cadherin-related functions or aberrant proliferation of NPCs, as probed in spinal cord [58]. In this study, we obtained results supporting the neurogenic function of Wnt signaling: overexpression of Tcf3 inhibits the canonical Wnt pathway and suppresses neuronal differentiation of neocortical NPCs under the same culture condition. Conversely, knockdown of Tcf3 promoted neuronal differentiation of the NPC culture. Moreover, Tcf3 expression is restricted to the undifferentiated NPCs expressing Pax6. We also found that expression of Tcf1 enhances the Wnt pathway and promotes neuronal differentiation. Therefore, these results regarding the functions of Tcf family members support the idea that Wnt promotes neuronal differentiation of neocortical NPCs.

The functions of the full-length Tcf3 in suppressing Wnt signaling and neuronal differentiation were similar to those of a Tcf3 mutant that lacks the N-terminal β-catenin binding domain, suggesting that the full-length Tcf3 acts as a repressor of Wnt signaling independently of its β-catenin binding. This feature of Tcf3 is commonly observed in other systems [23], [59]–[63] and recently in NPCs [34]. Importantly, Tcf3 was found to associate with the promoter of Neurog1 (and that of N-myc, [7]) and suppressed the expression of Neurog1 and N-myc (Fig. 4). Tcf3 has also been shown to suppress Sox4 independently of the Wnt–β-catenin pathway, when it suppresses neuronal differentiation in the developing spinal cord [64]. Therefore, it is plausible that Tcf3 binds to and suppresses neurogenic genes to prevent premature differentiation of NPCs. Tcf3 may thus work as a “brake” which ensures the undifferentiated state of stem cell population in the developing neocortex/central nervous system.

If Tcf3 serves as a “brake” of neuronal differentiation, which signals release this brake upon neuronal differentiation? Even though it has been postulated that β-catenin binding converts Tcf3 from a transcriptional repressor into an activator [25], Tcf3 can act as a repressor even in the presence of high levels of active β-catenin (Fig. 2B) [34]. Since the immunostaining signal of Tcf3 is relatively high in Pax6^+^ cells but becomes low in Neurog2^+^ cells in the neocortical VZ (Fig. 1), a reduction of Tcf3 protein appears to take place upon neuronal fate commitment of neocortical NPCs. In this study, we found that the treatment of cultured NPCs with recombinant Wnt3a or GSK3 inhibitor, which activates the Wnt–β-catenin pathway, markedly reduces the level of Tcf3 mRNA within a few hours. This Wnt-induced reduction of Tcf3 mRNA was accompanied by the reduction of Tcf3 protein. Therefore, Wnt signaling appears to utilize a positive feedback loop (in which Wnt activation downregulates its negative regulator Tcf3) for releasing the brake. Since Tcf3 protein has been shown to be evicted from its target loci by HIPK2-mediated phosphorylation (in Xenopus axis formation) [65] or by β-catenin binding (as mentioned above, [25]) in response to Wnt signaling, functional changes of Tcf3 in addition to the reduction of protein levels might also contribute to the derepression of Tcf3 target genes in response to Wnt signaling.

Other signaling molecules besides Wnt signaling may also regulate the level of Tcf3 and thereby the responsiveness of NPCs to Wnt signaling. Shh is likely to be among such signaling molecules, since the blockade of Shh signaling pathway by overexpressing a Patched mutant reduces, and the activation of this pathway by overexpressing an active Gli3 mutant increases, the level of Tcf3 mRNA in the developing chick spinal cord [66]. The crosstalk between Shh and Wnt signaling pathways is intriguing particularly because Shh signaling has been shown to promote proliferation (and maintenance) of NPCs.

In human intestinal epithelial cells, the most abundant Tcf1 isoforms lack the β-catenin binding domain, and Wnt signaling induces expression of these isoforms as a negative feedback [44]. Moreover, Tcf1^−/−^ mice develop adenomas in the gut, a typical phenotype induced by Wnt signal activation, suggesting that the repressor types are functionally dominant among Tcf1 isoforms in this tissue [44], [67]. However, in this study, we found that the full-length isoform of Tcf1 that contains the β-catenin binding domain is abundantly expressed in the developing mouse neocortex, and that stimulation with a Wnt ligand or a GSK3 inhibitor induces expression of this isoform. Given that overexpression of this full-length isoform enhanced the Wnt reporter transcription (Fig. 2) as reported before [24], [38], this Wnt-mediated induction of Tcf1 constitutes a positive feedback for Wnt signaling. We also found that Wnt ligand stimulation or treatment with GSK3 inhibitor induces expression of the full-length isoform of Lef1, a well-established positive regulator of Wnt signaling [47], [68], [69], in neocortical NPCs. Wnt-induced Lef1 expression has been observed in many other systems. In ES cells, it was recently proposed that Lef1 expression is repressed by Tcf3 and that Wnt stimulation releases this repression by β-catenin binding to Tcf3 [25]. In neocortical NPCs, the reduction of Tcf3 expression might thus contribute to the Wnt induction of Lef1 expression at least in part. Together, our study suggests that Wnt ligand stimulation triggers multiple positive feedback loops by suppressing its inhibitor (Tcf3) and by inducing its activators (Tcf1 and Lef1) in the neocortex.

These positive feedback loops may establish so-called “bistablen states of either Wnt-low or Wnt-high state [70]. In the Wnt-low state, Tcf3 might ensure that NPCs do not easily undergo neuronal differentiation in response to subthreshold levels of Wnt ligand stimulation. The positive feedback mechanisms might override the Wnt-low state and contribute to establishing the Wnt-high and irreversible differentiating state of NPCs. The further study of the trigger for the transition of NPCs from the Wnt-low to the Wnt-high state should provide new insights into the mechanism regulating the Wnt-induced differentiation of various types of stem cells.

## Supporting Information

Figure S1 **FACS sorting of Nestin-d4-Venus positive fractions. (Supplementary to** **Fig. 1I** **)** Dissociated cells from E14.5 neocortices of wild-type mice (above) or Nestin-d4-Venus transgenic mice (bellow) were analyzed by FACS and sorted into Nestin-d4-Venus−, Nestin-d4-Venus +, Nestin-d4-Venus ++, and Nestin4-Venus +++ fractions.
(TIF)

Figure S2 **Tcf3 does not so much affect the mRNA level of Cyclin D1 in NPCs. (Supplementary to** **Fig. 4** **)** ***A***, NPCs were infected with a retrovirus encoding control, Tcf3 shRNA #1 or Tcf3 shRNA #2 and incubated with FGF2 for 3 d. Cells were cultured for another 6 h in the absence of FGF2 (differentiated condition). The mRNA levels of Cyclin D1 was determined by qPCR analysis. ***B***, E11.5 NPCs were infected with a retrovirus encoding control or Tcf3 and incubated with FGF2 for 3 d. The level of Cyclin D1 mRNA was determined by qPCR analysis. ***A,B***, Data represents mean ± SEM.
(TIF)

Text S1 **Primers used in this study.**
(DOCX)
