# Supplementary figures and images for: Tcf3 Represses Wnt–β-Catenin Signaling and Maintains Neural Stem Cell Population during Neocortical Development
Source: PLoS One. 2014 May 15;9(5):e94408. doi: 10.1371/journal.pone.0094408 (PMC4022625; doi:10.1371/journal.pone.0094408)

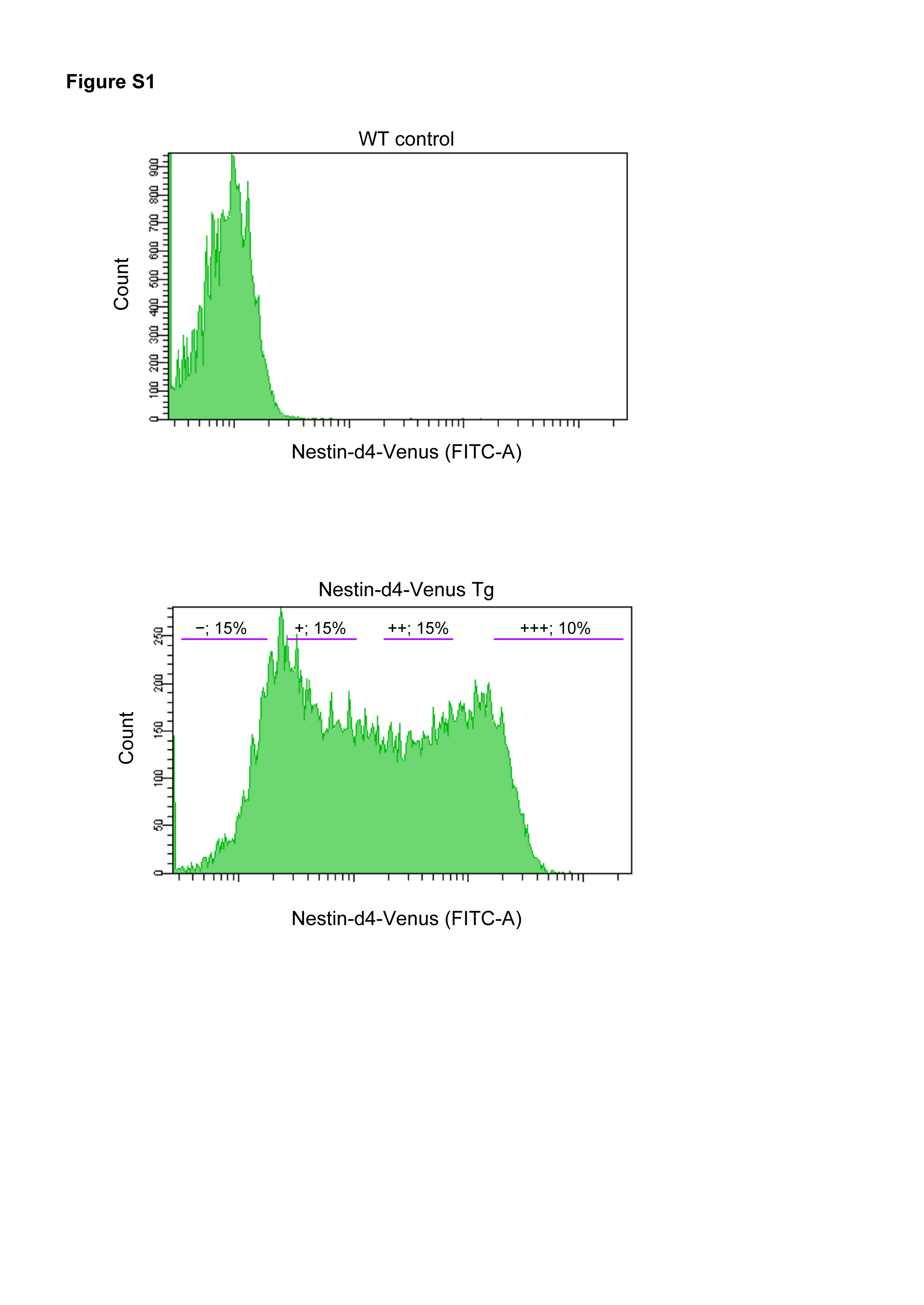

Supplement: Figure S1 — FACS sorting of Nestin-d4-Venus positive fractions. (Supplementary to Fig. 1I ) Dissociated cells from E14.5 neocortices of wild-type mice (above) or Nestin-d4-Venus transgenic mice (bellow) were analyzed by FACS and sorted into Nestin-d4-Venus−, Nestin-d4-Venus +, Nestin-d4-Venus ++, and Nestin4-Venus +++ fractions. (TIF) [file pone.0094408.s001.tif]

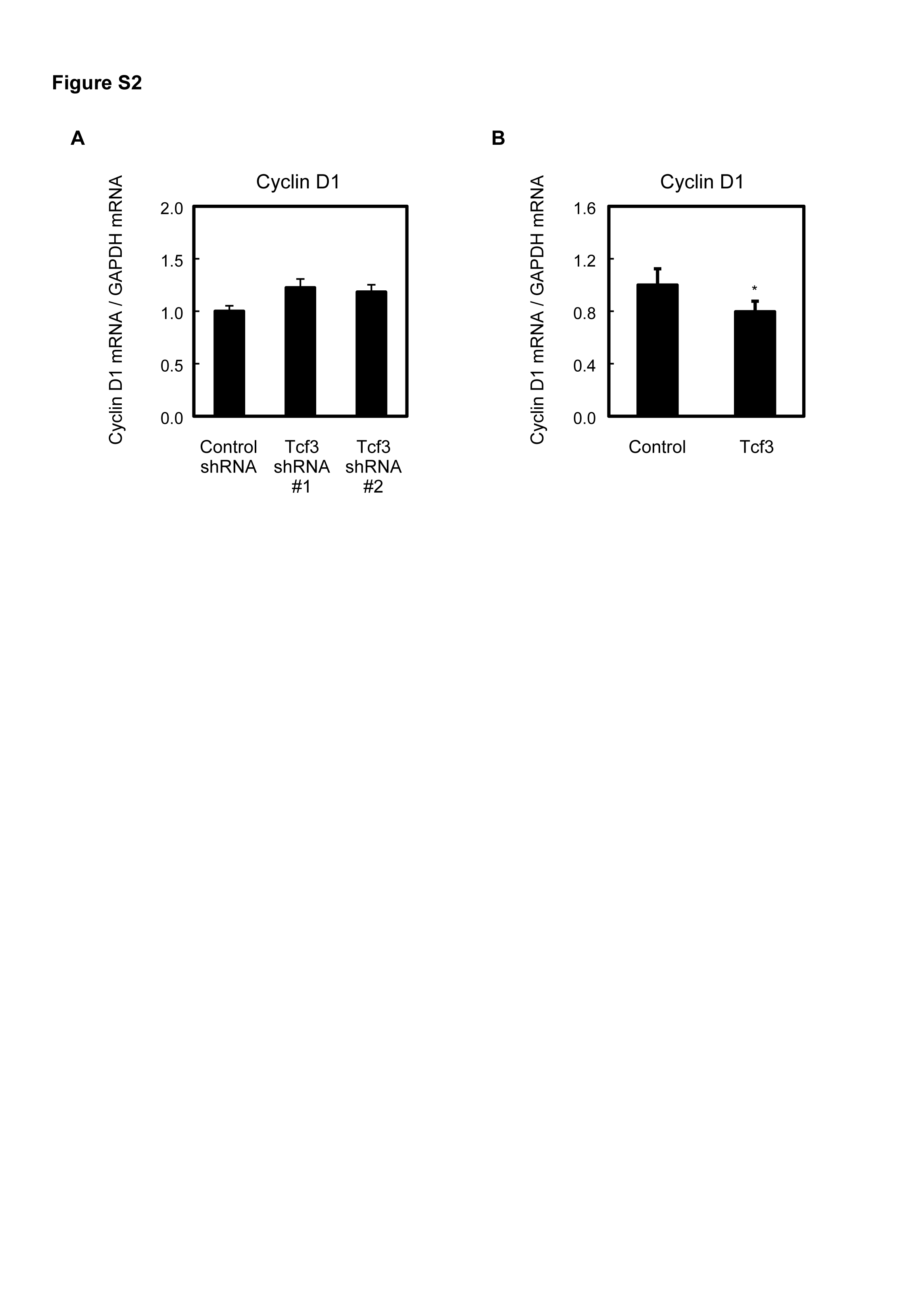

Supplement: Figure S2 — Tcf3 does not so much affect the mRNA level of Cyclin D1 in NPCs. (Supplementary to Fig. 4 ) A, NPCs were infected with a retrovirus encoding control, Tcf3 shRNA #1 or Tcf3 shRNA #2 and incubated with FGF2 for 3 d. Cells were cultured for another 6 h in the absence of FGF2 (differentiated condition). The mRNA levels of Cyclin D1 was determined by qPCR analysis. B, E11.5 NPCs were infected with a retrovirus encoding control or Tcf3 and incubated with FGF2 for 3 d. The level of Cyclin D1 mRNA was determined by qPCR analysis. A,B, Data represents mean ± SEM. (TIF) [file pone.0094408.s002.tif]
