# Supplementary material for: Tcf3 Represses Wnt–β-Catenin Signaling and Maintains Neural Stem Cell Population during Neocortical Development
Source: PLoS One. 2014 May 15;9(5):e94408. doi: 10.1371/journal.pone.0094408 (PMC4022625; doi:10.1371/journal.pone.0094408)
Supplement: Text S1 — Primers used in this study. (DOCX) [file pone.0094408.s003.docx]

**Text S1. Primers.**

Primers used for qPCR analysis were as follows;

GAPDH

5′-CTGAACGGGAAGCTCAC-3′

5′-GTCATCATACTTGGCAGGT-3′

Tcf3 (recognize both short form and long form)

5′-TGAAGGAAAGTGCAGCCATTA-3′

5′-TTTCCGGGCAAGCTCAT-3′

Tcf3-long form

5′-CCTCCAGTGCTACAGTCA-3′

5′-TGAATGTGGGAGGCACC-3′

Tcf3-short form

5′-GCACACTTGGTCCAACAAA-3′

5′-GAAGTGGTCGTTGCTGTAG-3′

Tcf4

5′-TTTGATGAGGGATTGGGAAG-3′

5′-AGACCATATTCTGCCTGTTG-3′

Tcf1 (1st-2nd exon)

5′-TCTCCATGTACTCGGACG-3′

5′-CAAGTCCTCACTGGTGAA-3′

Lef1 (1st-2nd exon)

5′-GCAGCTCTTTGCTTTGAC-3′

5′-CCCGATCCCTCTTTGTTC-3′

Neurog1

5′-ATCACCACTCTCTGACCC-3′

5′-GAGGAAGAAAGTATTGATGTTGCCTTA-3′

N-myc

5′-AATCGATGTGGTCACCGTA-3′

5′-GTTGTGCTGCTGATGGA-3′

For northern blotting and in situ hybridization analysis, PCR fragments were synthesized by using the sense and antisense primers as follows;

Tcf3

5′-AGCAGTAGCTCGGACTCCGA-3′

5′-AATTAACCCTCACTAAAGGGTGTCCGACAGCTCCTGGAGA-3′

Tcf1 (3rd exon)

5′-ATGCCGCAGCTGGACTCGGG-3′

5′-AATTAACCCTCACTAAAGGGGGCCTTCAGGCCGTCCTCTA-3′

Lef1 (2nd-5th exon)

5′-GGCCTTCAGGCCGTCCTCTA-3′

5′-AATTAACCCTCACTAAAGGGACCAGCCAATGGGTGGGGTGAT-3′

Primers used for ChIP assay were as follows;

*Neurog1* -1.6kbp (Tcf binding element)

5'-CAGAGGGAAGGAGCCAC-3'

5'-CCTAGCCTGCTGTCTTCA-3'

*Neurog1* TATA box

5'- CATTGTTGCGCGCCGTA -3'

5'- GCGATCAGATCAGCTCCT -3'

To generate Tcf3-ΔHMG, PCR fragments were synthesized by using the sense and antisense primers as follows;

5'- GGGCTTTTTCACGTGAGGTTTC -3'

5'- GAAAAGCAGCTGTCACAGAC -3'
